# Constraining Stroke Order During Manual Symbol Learning Hinders Subsequent Recognition in Children Under 4 1/2 Years

**DOI:** 10.3389/fpsyg.2020.00500

**Published:** 2020-03-20

**Authors:** Emily Merritt, Shelley N. Swain, Sophia Vinci-Booher, Karin H. James

**Affiliations:** Department of Psychological and Brain Sciences, Indiana University, Bloomington, IN, United States

**Keywords:** writing, development, children, early elementary education, symbol learning

## Abstract

In the age of technology, writing by hand has become less common than texting and keyboarding. Learning letters by hand, however, has been shown to have profound developmental importance. One aspect of writing by hand that has been understudied is the effect of learning symbols stroke-by-stroke, a dynamic action that does not occur with keyboarding. We trained children to draw novel symbols in either an instructed stroke order or in a self-directed stroke order and tested: (1) whether learning novel symbols in a self-directed stroke order benefits subsequent recognition more than learning in a specified stroke order, (2) whether seeing novel symbols unfold in the stroke order that was taught would aid in recognition, and (3) whether any effects are age-dependent. Our results demonstrate that producing a symbol with a self-directed stroke order provides more benefit to symbol recognition than instructed stroke orders in 4.0–4.5-year-old children but not in 4.5–5.0-year-old children. We found, further, that the observed recognition benefits were not affected by seeing the symbol unfold in the same stroke order it was learned during testing, suggesting that the learning was not reliant upon the exact stroke order experienced during learning. These results stress the importance of allowing children to produce symbols in a self-directed manner and, by extension, that constraining how a child learns to write can adversely affect subsequent recognition.

## Introduction

As more children and adults produce letters and words through texting or keyboarding, the day-to-day production of text by hand has diminished. The effect that this increased reliance on technology has on early language development is not well understood. We do know, however, that practicing writing letters by hand enhances letter recognition in children more so than learning the same letters by typing them (Longcamp et al., 2005) or by only seeing them (Zemlock et al., 2018). These findings have led researchers to stress the importance of handwriting during the letter-learning process (Berninger et al., 1997; Longcamp et al., 2005; James and Engelhardt, 2012; James et al., 2015; James, 2017). Why writing letters by hand improves letter recognition ability and, in turn, reading acquisition (Berninger et al., 1997, 2006) is still a topic of debate. There are several differences between typing a letter and producing it by hand that could lead to the observed benefit. For instance, the motoric requirements of the fingers, hand and wrist are very different when one produces a letter by hand compared with typing. In addition, there are visual differences – producing letters by hand results in viewing different forms of the same letter. This is especially true when *children* produce letters by hand – variability among produced instances of the same letter is high. Learning highly variable instances of symbols has been shown to be beneficial, compared with learning less-variable instances, for symbol categorization in 5-year-old children (Li and James, 2016). Another non-mutually exclusive idea is that seeing the letter unfold over time (compared with the complete form appearing at once) as it is being written allows the learner to focus on important features used for subsequent recognition.

This latter idea stresses the potential importance of dynamic information that can be perceived when a symbol is produced by hand – or when watching someone else produce a symbol. The stroke-by-stroke production of letters is an obvious omission when letters are typed. A series of elegant studies showed that knowledge of dynamic information that occurs during handwriting is used during the recognition of static letters (Freyd, 1983), enhances the ability to answer questions about imagined letters (Zimmer, 1982), influences symbol reproduction (Babcock and Freyd, 1988), and, in the case of cursive letters, the prediction of which letter will follow the previous (Orliaguet et al., 1997). These studies suggest that how a symbol is produced is stored and subsequently used to aid in visual recognition. That is, the strokes that are used to construct a symbol by hand may be highlighting important features or may be a part of the symbol representation itself.

The study described here was an initial attempt at addressing whether the way in which strokes are produced during novel symbol learning is an important aspect of subsequent recognition. We were interested in three major questions regarding the potential importance of stroke production. First, whether or not learning novel symbols in a specified stroke order benefits recognition more than learning in a self-directed stroke order. Second, we were interested in whether or not seeing novel symbols unfold in the stroke order that was taught would aid in recognition. If knowledge of how a symbol was drawn can influence recognition when it is shown in static form (Freyd, 1983), then it seems likely that introducing a specific stroke order, or drawing method, could also play a role when a symbol is shown stroke-by-stroke. Learned stroke order could influence recognition by providing a distinctive feature that, like the static features such as midsegments, terminals, and vertices (Gibson and Gibson, 1955; Gibson et al., 1962), becomes integral in the recognition process. To address this issue, we tested symbol recognition by displaying the symbols learned in a ‘stroke by stroke’ manner and manipulated learned vs. unlearned stroke orders. Because the participants only had to say whether the symbol itself was learned or unlearned, the potential influence of stroke order was implicit. Further, it has been found that the beneficial effects of handwriting on letter recognition are different in 4.5-year-old children than in younger children (Longcamp et al., 2005), suggesting that the window of time during which handwriting benefits letter recognition may be narrow and age-dependent. Therefore, we hypothesized that effects in this study may also be age-related and chose to investigate the window of time immediately before and after 4.5 years of age.

## Materials and Methods

### Participants

Forty-eight children between 4 and 5 years of age (*M* = 4.57, *SD* = 0.30, 28 males, two left-handed) participated in this study and received a small prize as compensation. Children were subsequently split into younger and older age groups to allow more sensitivity to age-dependent benefits of handwriting on letter recognition (Longcamp et al., 2005). A more detailed breakdown of the distribution of children is provided in Table 1. Although 56 children were originally recruited and took part in the study, some were excluded because they were unable to follow the instructions during our initial practice sessions (*n* = 8, 5 males). Children were recruited from a local database and contacted by phone and e-mail and were rewarded with a small toy for their participation. The parents of all participating children gave informed consent and the study was approved by the Institutional Review Board at Indiana University.

**TABLE 1:** Distribution of participants among age group and training condition.

		Training condition	
		Self-directed	Instructed
Age group	Younger	*n* = 15 *M* = 4.35 years, *R* = 4.06–4.59 years 10 Males	*n* = 11 *M* = 4.32 years, *R* = 4.05–4.57 years 5 Males
	Older	*n* = 11 *M* = 4.91 years, *R* = 4.64–5.04 years 6 Males	*n* = 11 *M* = 4.76 years, *R* = 4.62–4.92 years 7 Males

### Design

**FIGURE 1:**
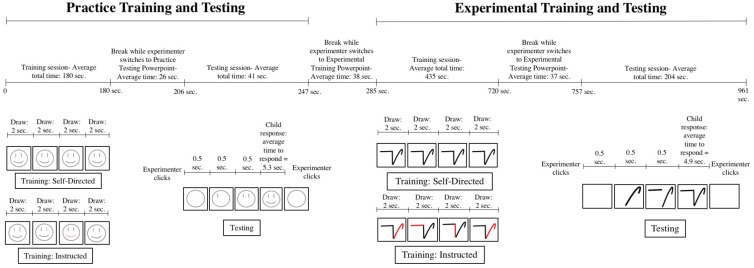
Example of the timeline of the study. Draw (in Practice and Experimental Training): child draws one stroke. During testing: The 0.5 s span refers to the amount of time between the appearance of each stroke (i.e., one stroke appears every 0.5 s until the entire face or symbol is on the screen). Child response: child answers “yes” or “no” as to whether they learned the face or symbol during training. Experimenter clicks: experimenter presses the space bar to initiate the Powerpoint and to advance it to the next face or symbol once the child has made their response. The stroke orders presented in the Practice Testing and Experimental Testing sections are merely examples and do not reflect the entirety of the stroke orders presented in the Testing Powerpoints; there were four types of testing stimuli presented in the Testing Powerpoint: learned faces or symbols (i.e., faces or symbols that the child practiced during training) presented in both a learned and an unlearned stroke order, and unlearned faces or symbols (i.e., faces or symbols that the child did not practice during training) presented in two different stroke orders.

There were two parts to the study – practice followed by the experimental session. Both parts were split into training and testing sessions (Figure 1). Children drew the stimuli during the training portions and performed a recognition test during the testing portions. The purpose of Practice Training and Testing was to familiarize the children with the experimental procedure and assess their ability to follow instructions and understand the directions necessary to complete the Experimental Training and Testing. No practice data were analyzed. The stimuli used during Practice Training and Testing were smiley faces; these are described in more depth in the “Stimuli” section below. A child’s successful completion of the Practice Training and Testing was assessed by whether or not they were willing/able to draw the stimuli one stroke at a time (in the case of the self-directed group) or whether they were willing/able to draw only the colored strokes (in the case of the instructed group). While all children completed Experimental Training and Testing, the data of children who failed to successfully complete Practice Training and Testing were excluded (8 total; 4 self-directed and 4 instructed see below).

Children were randomly assigned to either the self-directed or instructed training condition. Children in the self-directed group were told to choose the order in which they produced the strokes while children in the instructed group were given a specific stroke order to follow. Two symbol sets were included that were randomly and evenly assigned to individual children, but these were not analyzed as a variable of interest. Within the instructed group, we included a within-subjects variable that was manipulated in the recognition test; some trials included learned symbols (i.e., symbols that children drew during training) unfolding in the stroke order they were instructed to follow during training (learned order) and other trials included learned symbols unfolding in a stroke order they did not draw during training (unlearned order).

Experimental Training and Testing was performed with a novel symbol set that is described in more depth in the “Stimuli” section below. The primary dependent measure for all participants was correct or incorrect responses as to whether a symbol was learned during training or not (old/new symbol recognition). We also analyzed the symbol production phase of experimental training to assess the stroke orders chosen by children in the self-directed group.

### Materials

#### Stimuli

All stimuli were created and presented on an Apple Macbook Pro computer using Microsoft Powerpoint. Each stimulus was centered in the slide and shown against a white background.

##### Practice stimuli

**FIGURE 2:**
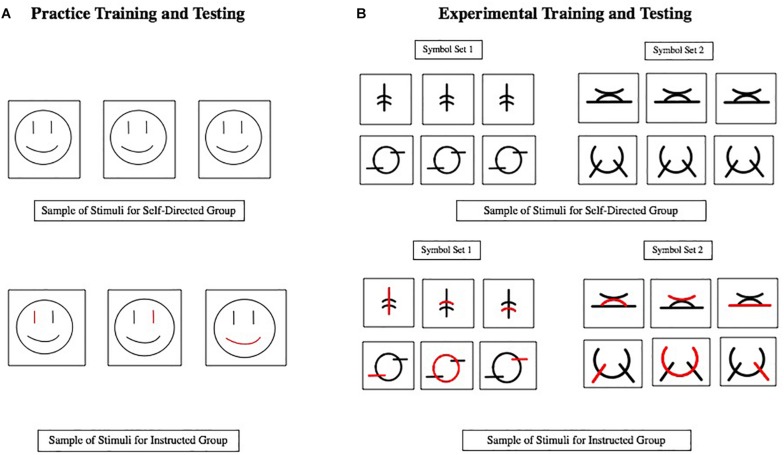
Examples of stimuli used in the practice training and testing **(A)** and experimental training and testing **(B)**.

During the practice session, stimuli consisted of three-stroke smiley faces (Figure 2A). The Practice Training Powerpoint slideshow consisted of eighteen stimulus slides and one blank slide at the start of the slideshow. Children in the self-directed group saw faces with only black strokes. Children in the instructed group saw faces with two black strokes and one red stroke. The red stroke indicated the stroke that the child was currently expected to draw. The stroke shown in red, therefore, changed on each slide. During the Practice Testing phase, children were required to respond to a presentation of a face that was revealed one stroke at a time in black ink only. There were eight presentations of faces in the testing phase. Two of the faces were learned during Training and were shown in two different stroke orders (one learned in training by the instructed group and one novel). The other four faces were novel and differed from the learned faces by either the eyes, mouth, or both; all were shown in one stroke order.

##### Experimental stimuli

Three-stroke novel symbols were used during the experimental sessions (Figure 2B). The Experimental Training Powerpoint was organized exactly like the Practice Training Powerpoint except that it presented novel symbols rather than faces.

The Experimental Testing session consisted of 32 slides that included eight symbols learned in training shown in two different stroke orders, one of which was the stroke order learned by children in the instructed group, and eight novel symbols shown in two different stroke orders. Therefore, there were 16 ‘old’ symbols and 16 ‘new’ symbols. All children were presented with the same 32 slides, but the order in which the slides were presented was randomized for each child.

#### Apparatus

Children were seated at a table facing an Apple Macbook Pro laptop computer, with the monitor at a distance of 47 cm. Children produced the faces and symbols with a colored marker on a 3″ × 5″ white index card. For Practice Training, both sides of each index card had a black circle 3 inches in diameter into which the child drew a face. For Experimental Training, the index card was blank on both sides.

A small Watec^TM^ brand camera was attached to a Velcro^TM^ band and wrapped around the child’s head such that the camera was on the center of their forehead; the camera angle was adjusted to give a view of only the child’s hands and index card. These recordings were collected to record the stroke orders selected during training by children assigned to the self-directed group; the data from the video recordings of children in the instructed group were not analyzed in this study (but they still wore the head camera). Additionally, each session was recorded by a camera mounted on the wall behind the child to afford a view of the child’s hands, the index cards, and the Powerpoint slide on the laptop.

### Procedure

The experiment began with Practice Training and Testing, in which children learned to draw two three-stroke faces by drawing each face three times in a row. Children in the self-directed group were instructed by the experimenter to draw each face one stroke at a time in an order of their choosing; after the child drew one stroke, the experimenter advanced the Practice Training Powerpoint to the next slide and the child drew another stroke. This continued until the child had drawn both faces three times. The only restriction placed on children was that one stroke be drawn per slide; children did not have to draw the same face in the same order each time that it was presented. Children in the instructed group, who saw a Practice Training Powerpoint that differed only from that seen by children in the self-directed group by the presence of the colored strokes described above, were instructed by the experimenter to draw only the red strokes. After the child completed the stroke, the experimenter advanced the Practice Training Powerpoint to the next slide. This continued until the child drew both faces three times. Following completion of the Practice Training slides, children in both the self-directed and instructed groups were then presented with the Practice Testing Powerpoint. Children were instructed to watch each face be drawn on the screen and then to verbally state “yes” or “no” or to shake their head up and down for “yes” or side to side for “no” when asked by the experimenter whether or not they had just drawn each face. Because each face appeared on the screen one stroke at a time, children were instructed to wait until the entire face (all three strokes) was present on the screen before responding. The experimenter advanced through the Powerpoint slides after the child answered and did not record their responses or provide feedback on the accuracy of their response.

Following completion of Practice Training and Testing, children immediately proceeded to Experimental Training and Testing. Each child was shown the appropriate training slides depending on their assigned symbol set and group. As in the Practice Training, the only difference between the Experimental Training Powerpoints seen by children in the self-directed and instructed groups was the inclusion of colored strokes to dictate stroke order to children in the instructed group. Experimental Training proceeded in the same manner as Practice Training. Upon completion of Experimental Training, children immediately moved on to Experimental Testing. The experimenter initiated the randomized Experimental Testing Powerpoint and continued with Experimental Testing exactly as they did with Practice Testing, except that in this instance they recorded the child’s response by hand, noting if the child changed their answer, was distracted during the presentation of the symbol, or took a long time to answer. As in Practice Testing, children were instructed to respond only after the entire symbol (all three strokes) had appeared on the screen, and only responses given after the full symbol was visible were counted. Children again did not receive feedback on their response. A correct answer was for the symbol itself, not the stroke order. For instance, a correct answer was attained when a child either said “yes” to a symbol that they had learned or “no” to a symbol that they did not learn, regardless of what stroke order the learned symbol was shown. During testing, all children were presented with each symbol twice, each in a different stroke order. Each time the symbol unfolded stroke by stroke and the entire stroke appeared as a whole immediately rather than being drawn on the screen. For children in the instructed group, one of the stroke orders (the “learned” stroke order) matched the order in which they drew the symbol during Experimental Training; the second stroke order was novel (the “unlearned” stroke order). Children in the self-directed group were exposed to these same two stroke orders, and the video recordings were analyzed to assess whether or not the two stroke orders presented during Experimental Testing matched the stroke orders that were chosen during Experimental Training.

## Results

### Self-Directed vs. Instructed Stroke Orders During Training

**FIGURE 3:**
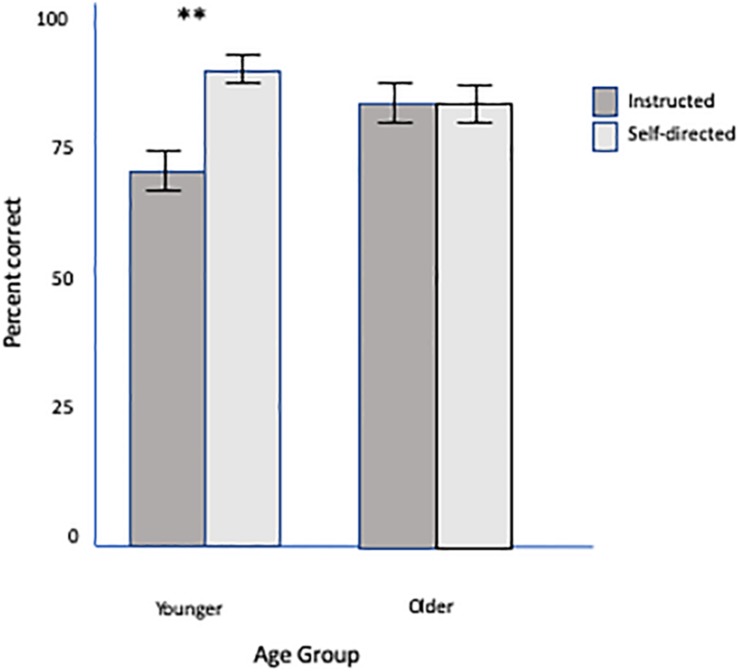
Interaction of condition × age group. Mean percent correct as a function of age group. Error bars reflect standard error of the mean. **p < 0.005.

Children in the self-directed group performed better on the recognition test compared to children in the instructed group. This effect, however, was only observed in the under 4.5-year age group. A two-way analysis of variance (ANOVA) with stroke (self-directed, instructed) and age (under 4.5 years, over 4.5 years) as between-subjects factors and proportion of correct trials as the dependent variable revealed a significant main effect of stroke condition [*F*(1,44) = 5.49, MS = 0.085, *p* < 0.05], a significant main effect of age [*F*(1,44) = 4.47, MS = 0.070, *p* < 0.05], and a significant interaction between stroke condition and age [*F*(1,44) = 5.13, MS = 0.080, *p* < 0.05]. The interaction was a result of greater overall accuracy for the younger age group in the self-directed condition (*M* = 0.88, *SD* = 0.11) compared with the instructed condition (*M* = 0.72, *SD* = 0.15), [*t*(24) = 3.32, *p* < 0.005], but no difference between the two stroke conditions in the older group [*t*(20) = 0.06, *p* = ns] (Figure 3).

Children in the self-directed condition were free to select their own stroke order for each symbol. It is possible that they selected the same stroke orders as they would have experienced in the instructed condition, suggesting that the recognition benefit for self-directed over instructed stroke orders reported above was simply related to the absence of instruction. We measured the average number of times a child in the self-directed group selected a stroke order that was the same as what would have been the instructed. Symbols were drawn 24 total times during training. Younger children in the self-directed group selected stroke orders that were the same as what would have been instructed on average 18.89% (*SD* = 9.8) of the time [one-sample *t*-test; *t*(14) = −31.99, *p* < 0.001]. Older children in the self-directed group selected stroke orders that were the same as what would have been instructed on average 17.75% (*SD* = 12.6) of the time [one-sample *t*-test; *t*(10) = −21.65, *p* < 0.001]. This suggests that the benefit of self-directed over instructed stroke orders on recognition is not likely related to the absence of instruction alone.

### Learned vs. Unlearned Stroke Orders During Testing

Children in the instructed group were subjected to an additional manipulation that evaluated whether or not recognition at test would be affected by seeing the strokes of a learned symbol appear in learned compared to unlearned orders. We found no evidence that recognition is affected by seeing a symbol unfold as it did during learning. Younger children performed similarly at recognition testing, regardless of whether the stroke order was previously learned (*M* = 0.74, *SD* = 0.25) or not (*M* = 0.68, *SD* = 0.30). Older children also performed similarly at recognition testing, regardless of whether the stroke order was previously learned (*M* = 0.83, *SD* = 0.18) or not (*M* = 0.82, *SD* = 0.23). This finding was supported by a two-way ANOVA with age (under 4.5 years, over 4.5 years) as a between-subjects factor, stroke order (learned and unlearned) as a within-subjects factor and proportion of correct trials as the dependent variable. This yielded no significant main effect of age [*F*(1,20) = 1.36, MS = 0.142, *p* = ns] or stroke order [*F*(1,20) = 1.10, MS = 0.013, *p* = ns], and no interaction [*F*(1,20) = 0.49, MS = 0.006, *p* = ns]. This indicates that, in this study, watching strokes appear in a learned stroke order did not aid in recognition of novel symbols.

## Discussion

In this study, we examined how learning to produce novel symbols affected recognition in preschool aged children. We first tested whether or not learning to produce a symbol in a self-directed stroke order would facilitate recognition compared with producing a symbol in a specified stroke order. Our results demonstrated that recognition was better when children were able to self-select stroke order compared to children who were instructed to perform a specified stroke order. The recognition benefit from self-directed stroke orders only occurred in younger children (<4.5 years). Further, the self-selected stroke order was not the same as the instructed one (that was the typical up-to-down and left-to-right) in most cases. Second, we found that seeing the strokes of a novel symbol appear in a previously learned order during recognition did not affect recognition when compared to seeing the strokes appear in an unlearned stroke order in either age group. Together, these findings suggest that when first learning symbols, producing them in a self-directed stroke order helps recognition in young preschool children but that the stroke order information learned during production is not influential in subsequent recognition. These findings suggest that constraining the manner in which young children produce symbols by hand to a specific stroke order is not the most effective educational strategy in early preschool. This educational practice may hinder recognition and, therefore, have little to no benefit in early preschool relative to self-directed symbol production.

### Constraining Stroke Order During Symbol Production Hinders Recognition

Children are typically taught letters in particular stroke orders when learning letters through handwriting. Little attention, however, has been paid to the efficacy of these stroke orders in helping children learn to recognize letters. Our results suggest that teaching children a specific stroke order might actually reduce the effectiveness of handwriting for learning letters. This effect only occurred in our younger group of children, suggesting that self-directed production is most effective in young preschool children. There are many possible reasons why young preschool children may be hindered by stroke order instruction. It could be, for instance, that young preschoolers have more difficulty attending to several instructions at once (e.g., Engle et al., 1991; Fry and Hale, 2000; Gathercole et al., 2008). There were higher attentional demands placed on children in the instructed group; in contrast to their counterparts in the self-directed group, children in the instructed group were required to remember more rules and pay closer attention to what they were doing in order to correctly draw the symbol. For example, while children in both groups were instructed to learn the symbol and draw only one stroke at a time, those in the instructed group had the additional requirement of remembering that the red stroke was the one that they were to draw.

In addition, although the entire symbol was present on each training slide for all children, the appearance of one red-colored stroke may have distinguished that stroke too much, causing the children in the instructed group to see the symbol more as a series of distinct strokes rather than as a whole each time that they looked at it. Although this would seem to suggest, then, that children in the instructed group might better recognize learned symbols when they are shown in their learned stroke order as opposed to an unlearned order, we did not see this kind of memory benefit. This could be due to the nature of our test: we asked children to identify the symbol itself, not the stroke order that was used to create it. If children in the instructed group did focus more on the individual strokes as opposed to the symbol as a whole, they may be better able to recognize the process, or stroke order, used to create a symbol rather than the actual symbol itself. Future studies addressing identification of learned or unlearned stroke orders would be useful in further clarifying the role of stroke order in symbol recognition.

Another possible explanation is that children in the self-directed group produced the strokes in an order that was consistent with their own perceptual experience of the symbol. When presented with a symbol, a child in the self-directed group was free to produce whatever stroke seemed most accessible, whether this was simply the first stroke on which they fixated or the stroke that seemed easiest to produce or, perhaps, even the stroke that seemed most difficult to produce. Whatever the reason for their choice, they were free to make it. A child in the instructed group, however, likely still had the urge to produce that particular stroke but was made to focus elsewhere. In this way, the ability to execute a particular motor plan, or the actions necessary to accomplish a particular goal (Pennequin et al., 2010), was different depending on whether a child was assigned to either the instructed or self-directed group. When there is more than one conceivable way to complete that goal, the choice and execution of just one plan requires the inhibition of the others. The symbols used in this study were each composed of three strokes, introducing a variety of methods, or plans, that could all be used to accomplish the task of drawing the symbol one stroke at a time. In this way, upon seeing each symbol for the first time, it is likely that each child would have had a preferred stroke order, or plan, by which to draw the symbol; children in the self-directed group would have been able to follow that plan, whereas children in the instructed group may have been required to use a different plan.

Some research looking at motor planning and inhibition supports the idea that younger children may have more difficulty than older children in tasks that might require them to suppress their initial urge to respond in a particular way (Russell et al., 1991; Gerstadt et al., 1994; Pennequin et al., 2010). In each of these studies, researchers found that older children were better able to inhibit competing motor plans in favor of the one that best accomplished the task at hand; this result offers a potential explanation for the age-dependency of our finding that children in the instructed group performed worse on the recognition test than children in the self-directed group. It could be that the children in the instructed group were forced to inhibit the motor plan that they would have chosen to use had they been given the freedom to do so. This idea is supported by our evidence that children in the self-directed group chose stroke orders that were significantly different from those used by children in the instructed group. In this way, our results seem to suggest that children in the instructed group, who were given a specific plan, or stroke order, to use, were forced to draw a symbol using a motor plan that was not their preferred choice. However, we cannot be certain of this because we did not assess what stroke orders children in the instructed group would have chosen; in this study, our best measure of this comes from an analysis of the stroke orders chosen by children in the self-directed group. Our results, then, suggest that forcing children to inhibit their preferred motor plan (or, in this case, their use of a preferred stroke order) negatively affects their ability to distinguish between learned and unlearned symbols, and that this poses a more detrimental effect to recognition in younger children between 4 and 4.5 years of age as opposed to older children between 4.5 and 5 years of age.

### Learned Stroke Order Does Not Influence Symbol Recognition

We were unable to find any evidence to support the idea that stroke order information is important for recognition of novel symbols in 4.0–4.5-year-old or 4.5–5.0-year-old children. When comparing recognition between symbols unfolding in learned and unlearned stroke orders, we found no significant differences between symbols shown in learned and unlearned stroke orders in either age group. This result was somewhat surprising, given that several adult studies have suggested that the typical motion patterns of an object are influential in subsequent recognition of that object (Freyd, 1983; Babcock and Freyd, 1988; Orliaguet et al., 1997; Knoblich et al., 2002; Parkinson and Khurana, 2007). In addition, a recent case study suggested that the stroke orders learned during letter production might remain influential years after they are learned, though they are often masked by ceiling effects. This patient, who had an acquired deficit in letter recognition, was better at letter identification when the letters were presented unfolding stroke by stroke, but only when the unfolding occurred in the standard stroke order (Schubert et al., 2018). It is possible that stroke order information is more important for subsequent recognition in adults than in children.

Adults are experts at letter production and use an invariant stroke order relative to children. Children are still exploring how to produce symbols and, therefore, are more likely to vary their stroke order from one production to the next. The stroke order patterns in this study were far from being well-learned – perhaps leading to no benefit to recognition in these young children. Further studies are needed to more fully understand the role, if any, stroke order information has on symbol recognition in children.

## Conclusion

This study revealed that constraining stroke order via instruction during manual novel symbol learning hinders subsequent recognition in young children (<4.5 years). These results stress the importance of self-directed production of symbols on subsequent recognition and suggest that constraining the manner that young preschool children produce symbols by hand may, in fact, be a hindrance to learning to recognize letters. Unlike findings in adults, we were unable to find any evidence that stroke order is stored and influential during subsequent recognition in young children. These results suggest further, corroborating other research (e.g., Longcamp et al., 2005; Zemlock et al., 2018), that producing letters by hand has unique, age-dependent contributions to letter learning.

## Data Availability Statement

The datasets generated for this study are available on request to the corresponding author.

## Ethics Statement

The studies involving human participants were reviewed and approved by Indiana University Institutional Review Board. Written informed consent to participate in this study was provided by the participants’ legal guardian/next of kin.

## Author Contributions

KJ: PI of laboratory that supports the research, initiated original idea, supervised all other authors, edited the manuscript, and pays subjects and researchers. EM: head researcher of project, ran subjects, analyzed the data, and wrote the initial manuscript. SS: ran subjects, analyzed the data, and edited the manuscript. SV-B: analyzed the data, designed the experiments, and edited the manuscript.

## Conflict of Interest

The authors declare that the research was conducted in the absence of any commercial or financial relationships that could be construed as a potential conflict of interest.
